# Supplementary figures and images for: Dynamic kernel matching for non-conforming data: A case study of T cell receptor datasets
Source: PLoS One. 2023 Mar 7;18(3):e0265313. doi: 10.1371/journal.pone.0265313 (PMC9990938; doi:10.1371/journal.pone.0265313)

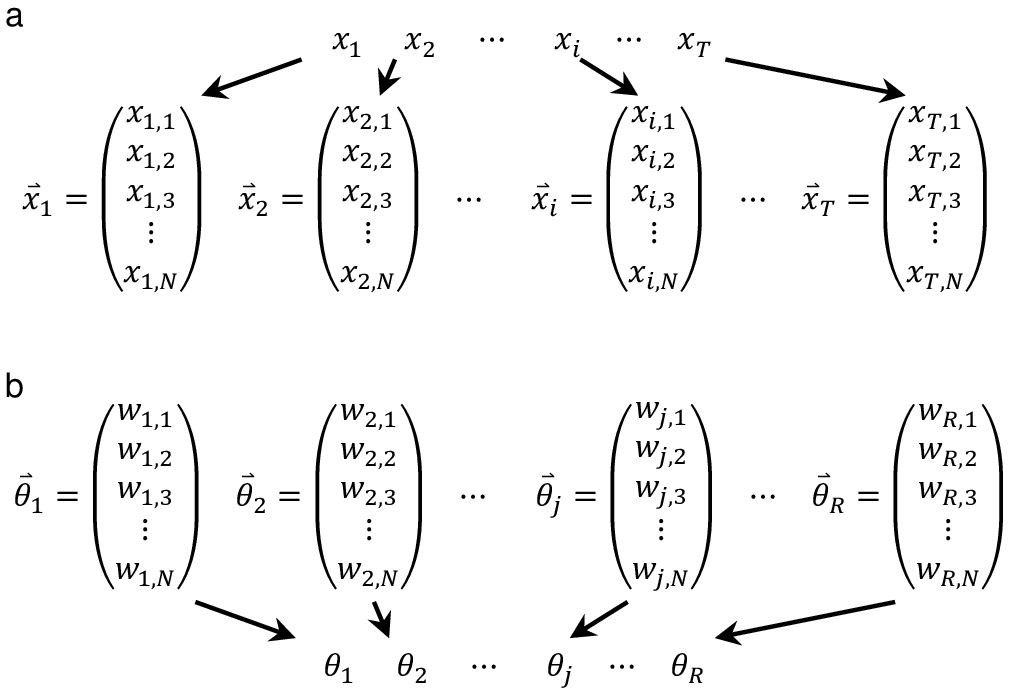

Supplement: S1 Fig — (a) Let us assume the non-conforming features consists of a sequence (or set) of T symbols x1, x2, … xi, ‥ xT. We replace each symbol with a vector of N numbers describing that symbol, resulting in a sequence (or set) of T vectors x⇀1,x⇀2,…x⇀i,…x⇀T. (b) Let us assume the number of weights for our statistical classifier can form R groups of N weights. Each group of N weights is used to form a vector, allowing us to write the weights as the vectors θ⇀1,θ⇀2,…θ⇀j,…θ⇀R. We can think of each vector as a symbol, forming the symbols θ1, θ2, … θj, … θR. (TIF) [file pone.0265313.s001.tif]

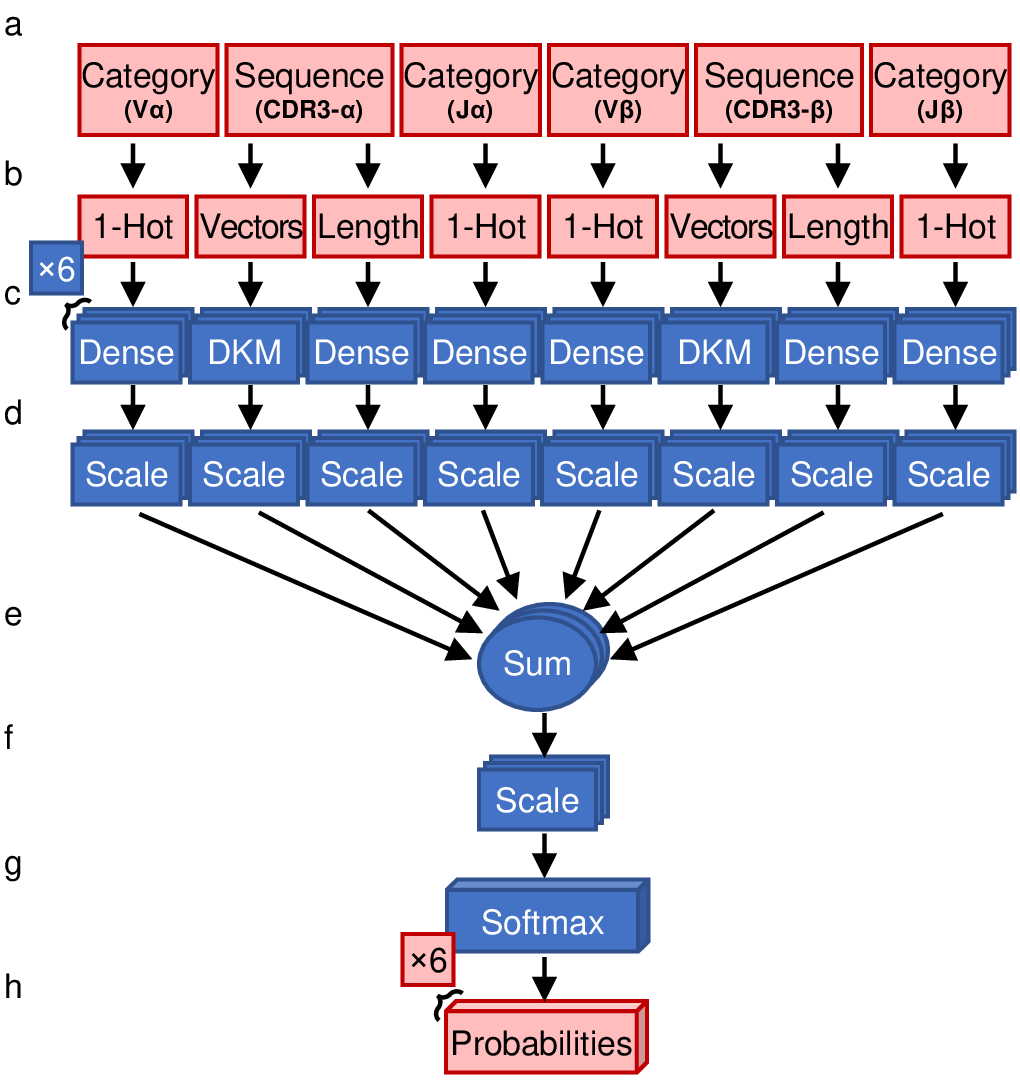

Supplement: S2 Fig — (a) Features for each TCR are partitioned into six groups. (b) The representations for each feature group. (c) Dense modules assign a weight to each feature and compute a dot product. The DKM modules use a sequence alignment algorithm to match features with weights and compute a dot product. (d) Each dot product is normalized over training samples. The layout is repeated six times, one for each category of label. (e) The scaled dot products are added together into one dot product. (f) The resulting dot product is normalized over training samples. (g) The dot product is part of a multinomial logit and passed through a softmax function. (h) The softmax function produces probabilities representing the predictions of the statistical classifier. The six probabilities, one for each category, always sum to one. (TIF) [file pone.0265313.s002.tif]

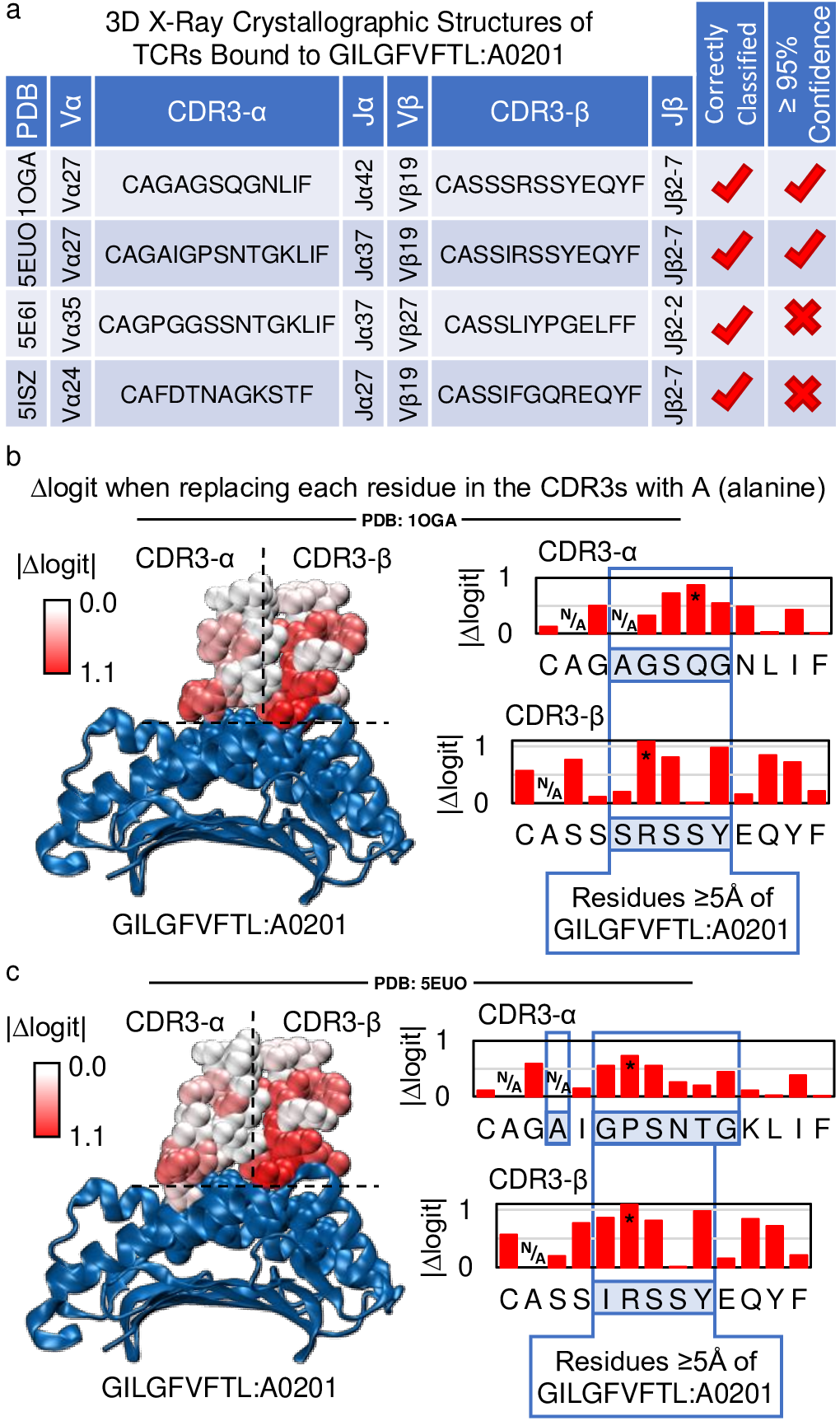

Supplement: S3 Fig — (a) Four 3D X-ray crystallographic structures found with a TCR bound to GILGFVFTL:A0201. All four TCRs are correctly classified and two with ≥95% confidence (see Applying Confidence Cutoffs). (b) An alanine scan of the 3D X-ray crystallographic structure 1OGA (four letter codes are the protein databank identifier at https://www.rcsb.org/) reveals the |Δlogit| tend to be greater (redder) for pMHC (green) contact positions (≤5Å) than non-contact positions. The largest |Δlogit| for each CDR3 is always a contact position. (c) Same as before for another 3D X-ray crystallographic structure 5EUO. (TIF) [file pone.0265313.s003.tif]

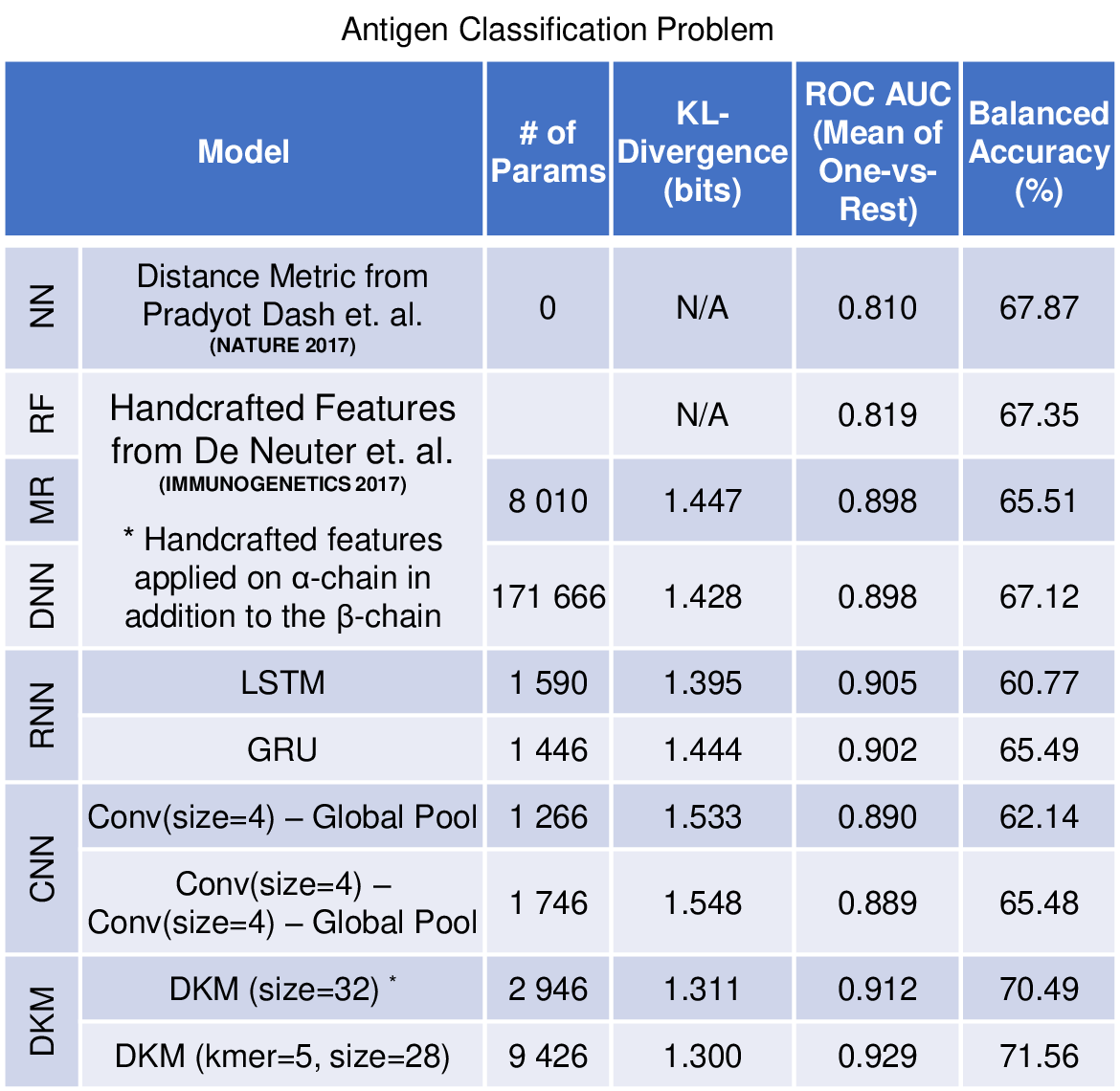

Supplement: S4 Fig — Performance is measured using (i) KL-divergence, (ii) area under the curve (AUC) for true over false positive rates, and (iii) balanced classification accuracies. All models are fitted to the training cohort and results reported on the test cohort. The considered models are NN (nearest neighbors), RF (random forest), MR (multinomial regression), DNN (deep neural network), RNN (recurrent neural network), CNN (convolutional neural network), and DKM (dynamic kernel matching). DKM models have the best KL divergence fit, the highest AUC, and the highest balanced classification accuracies over the test cohort. The DKM model with the asterisk is the model reported in the main text. We performed a post-hoc hyperparameter optimization of our DKM model and found that we could achieve better results classifying each CDR3 as a sequence of 5-mers. However, we had already unblindfolded ourselves to the test cohort, so we did not report this result in the main text. (TIF) [file pone.0265313.s004.tif]

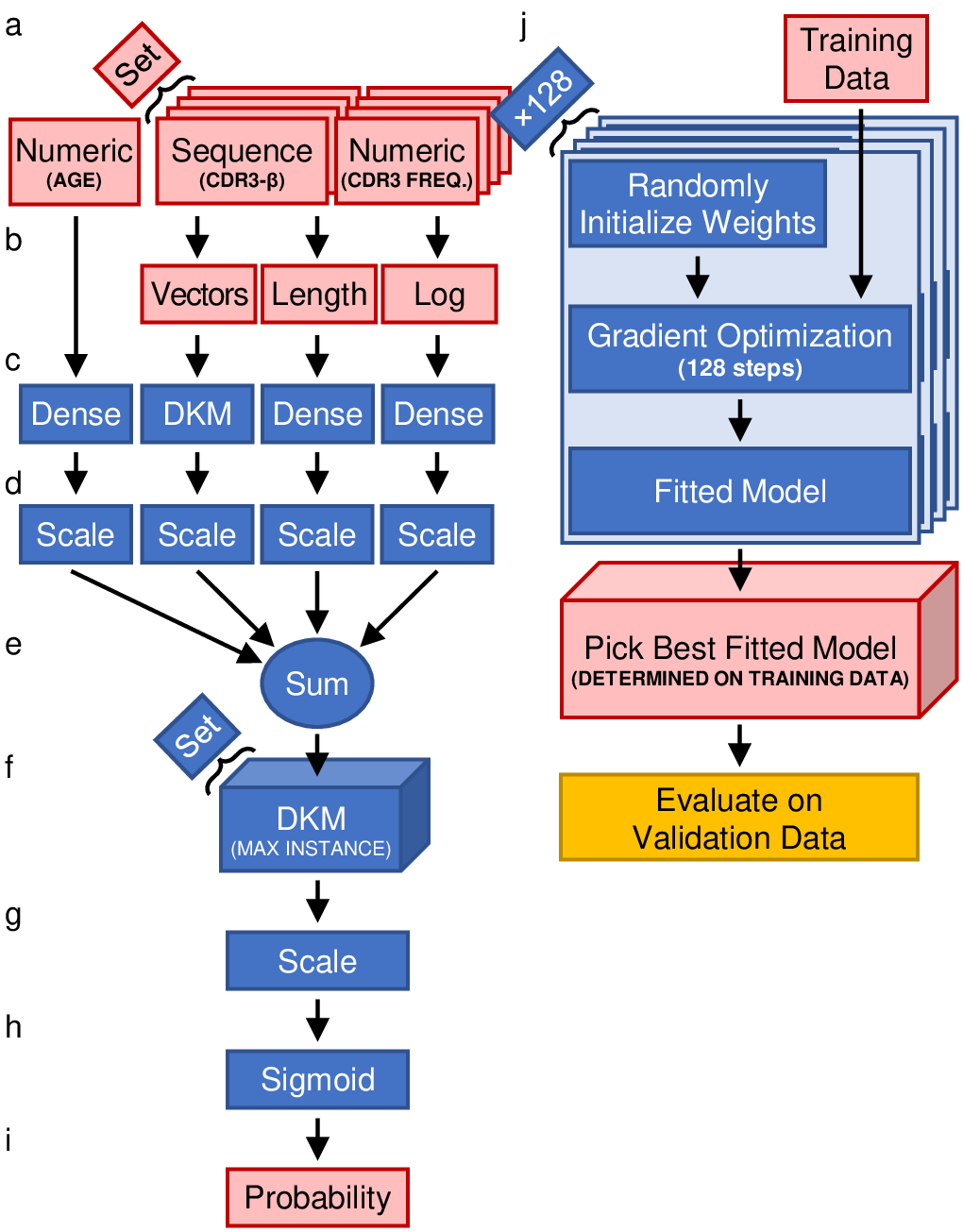

Supplement: S5 Fig — (a) Features for each patient are partitioned into three groups. The last two feature groups, representing the CDR3-β sequence and relative CDR3 frequency, form a set. Each member of the set is passed through the model. (b) The representations for each feature group. (c) Dense modules assign a weight to each feature and compute a dot product. The DKM modules use a sequence alignment algorithm to match features with weights and compute a dot product. (d) Each dot product is normalized over training samples. (e) The scaled dot products are added together into one dot product. (f) This DKM module takes the maximum value over the set, which is the computation required to solve the assignment problem for the special case where \Theta contains just one sequence (g) The maximum value is normalized over training samples. (h) The scaled value is passed through a sigmoid function. (i) The sigmoid function produces a probability representing the prediction of the statistical classifier. (j) Random values for each weight are refined by 128 steps of gradient optimization to produce a fitted model. Weights from the best of 128 attempts to fit the model are used to evaluate the model on the validation cohort and eventually the test cohort. (TIF) [file pone.0265313.s005.tif]

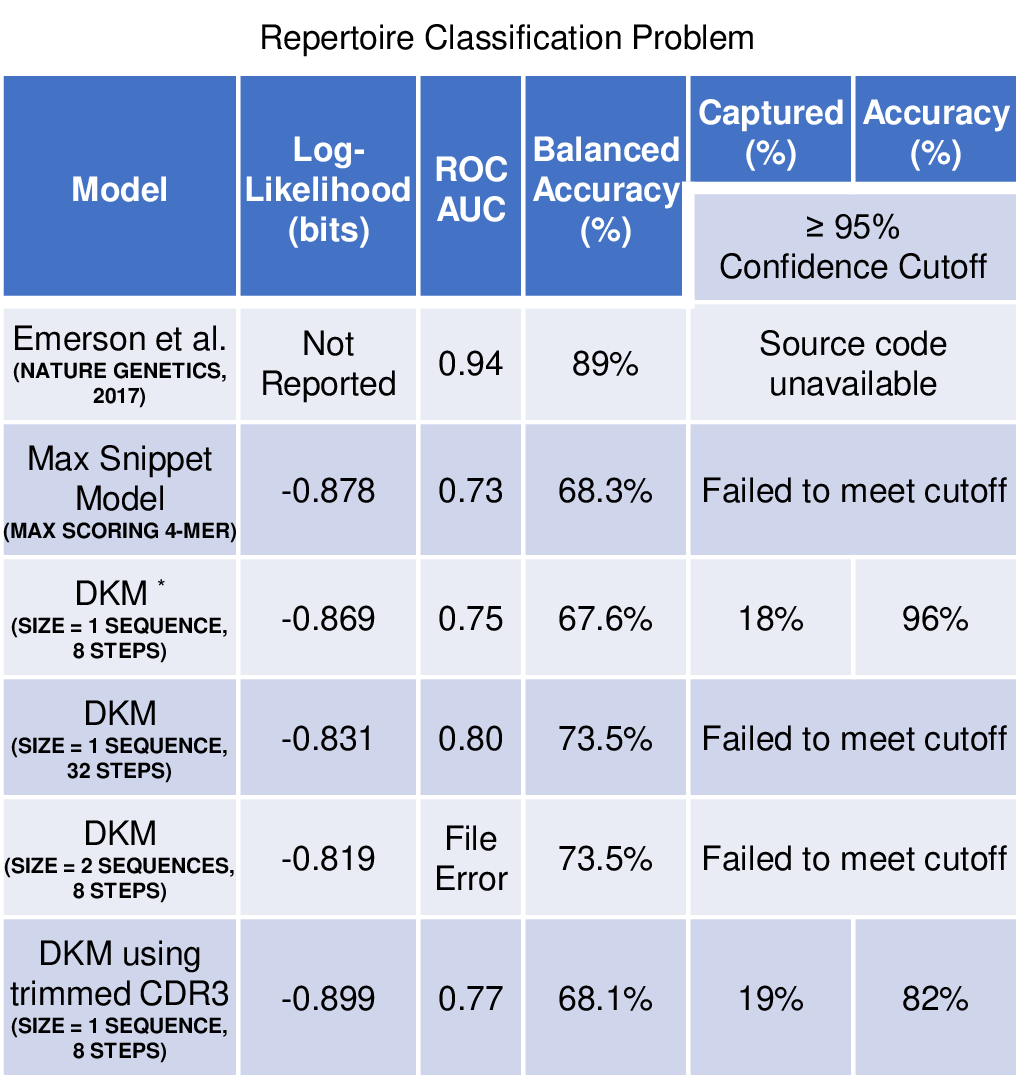

Supplement: S6 Fig — Performance is measured using (i) log-likelihood, (ii) area under the curve (AUC) for true over false positive rates, (iii) balanced classification accuracy, and (iv) confidence cutoffs. The approach published by Emerson et al. achieves the best classification accuracy. We could not calculate the log-likelihood fit or apply confidence cutoffs because the source code to their method is unavailable. Instead, we use the results reported in their publication. The Max Snippet Model, as described in our earlier publications, has the worst performance [1–3]. The asterisk indicates the model reported in the main text. Using confidence cutoffs, we achieve a classification accuracy of 96%, capturing 18% of patients. We also performed a post-hoc hyperparameter optimization of our DKM model (last three rows) and found that we could achieve better results when the weights are arranged into 32 steps instead of 8 and for another DKM model where the weights are arranged into two sequences instead of one. Both these DKM models achieve better performances on the test cohort than the DKM model we report in the main text. However, we had already unblindfolded ourselves to the test cohort, so we did not report these result in the main text. (TIF) [file pone.0265313.s006.tif]

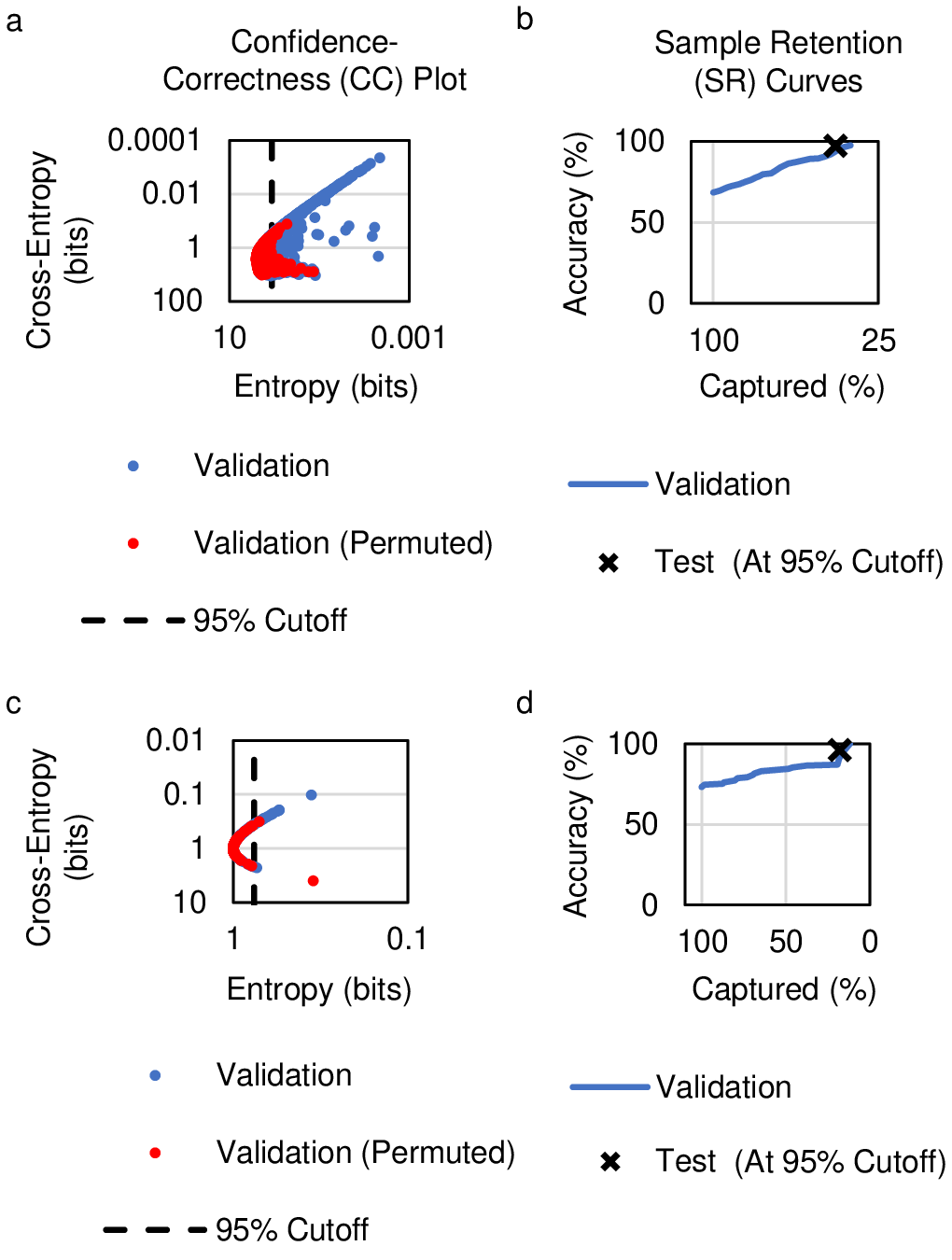

Supplement: S7 Fig — (a) Using entropy as a measure of confidence, which is computed without labels, and cross entropy as a measure of correctness, which is computed with labels, we see a correlation between the confidence and correctness over each prediction (blue dots) of the statistical classifier on the antigen classification problem. Because the correlation exists, we can use the confidence to enrich for correctness, where the former is computed without knowing the labels. Samples captured to the right of the dashed line are classified with >95% accuracy (Fig 4), while samples to the left are considered indeterminant. No correlation exists for permuted data (red dots). (b) Sample retention curves show the classification accuracy as the confidence cutoff is increased, reducing the number of captured samples. Using the 95% cutoff computed using the validation cohort, samples from the test cohort are captured and the accuracy computed (“x”). (c) Like panel a, for the repertoire classification problem. (d) Like panel b, for the repertoire classification problem. (TIF) [file pone.0265313.s007.tif]

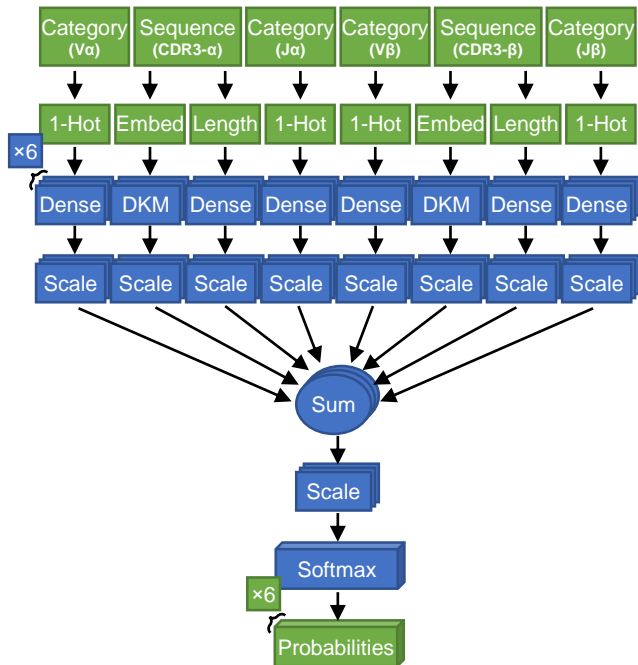

Supplement: S1 Data — (ZIP) [file pone.0265313.s009.zip › source code/artwork/antigen-classification-model.pdf]

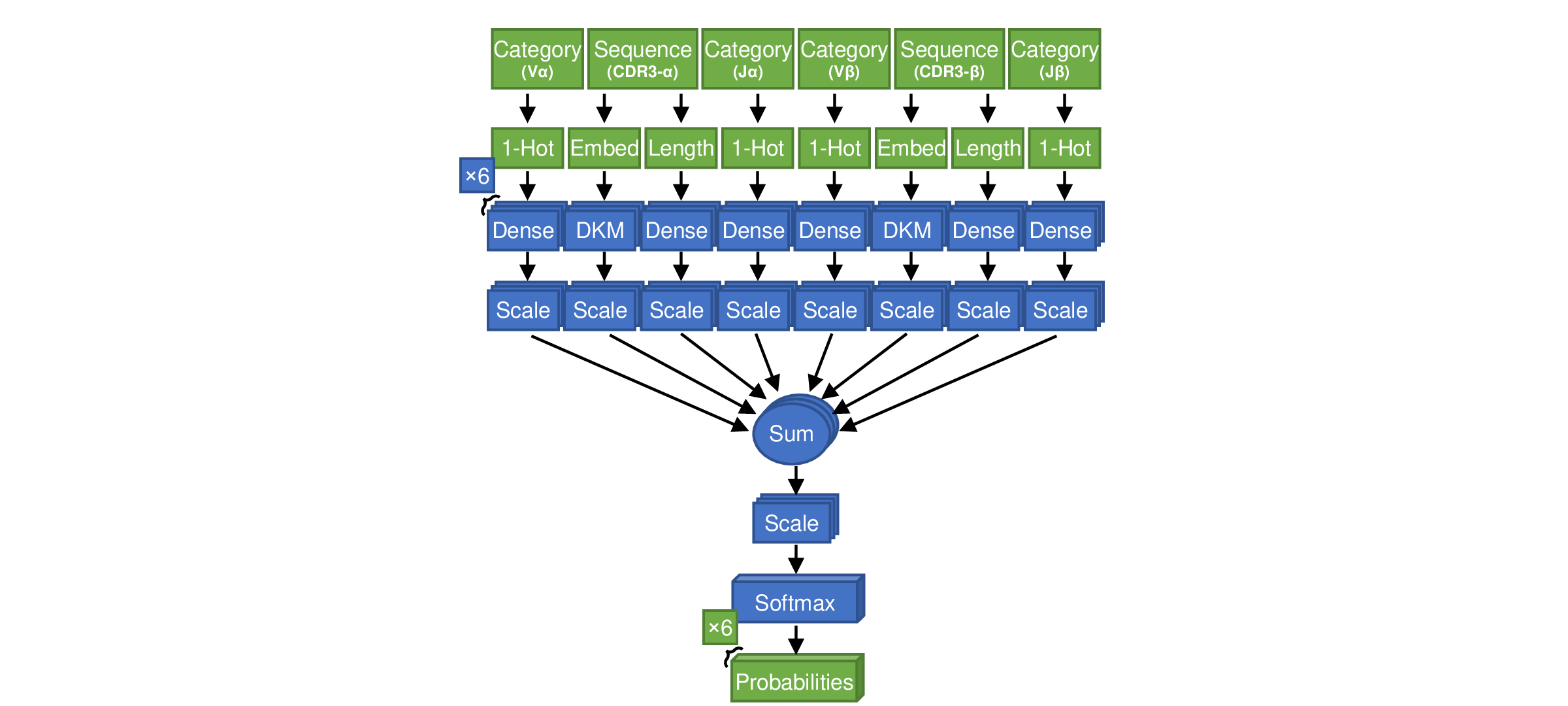

Supplement: S1 Data — (ZIP) [file pone.0265313.s009.zip › source code/artwork/antigen-classification-model.png]

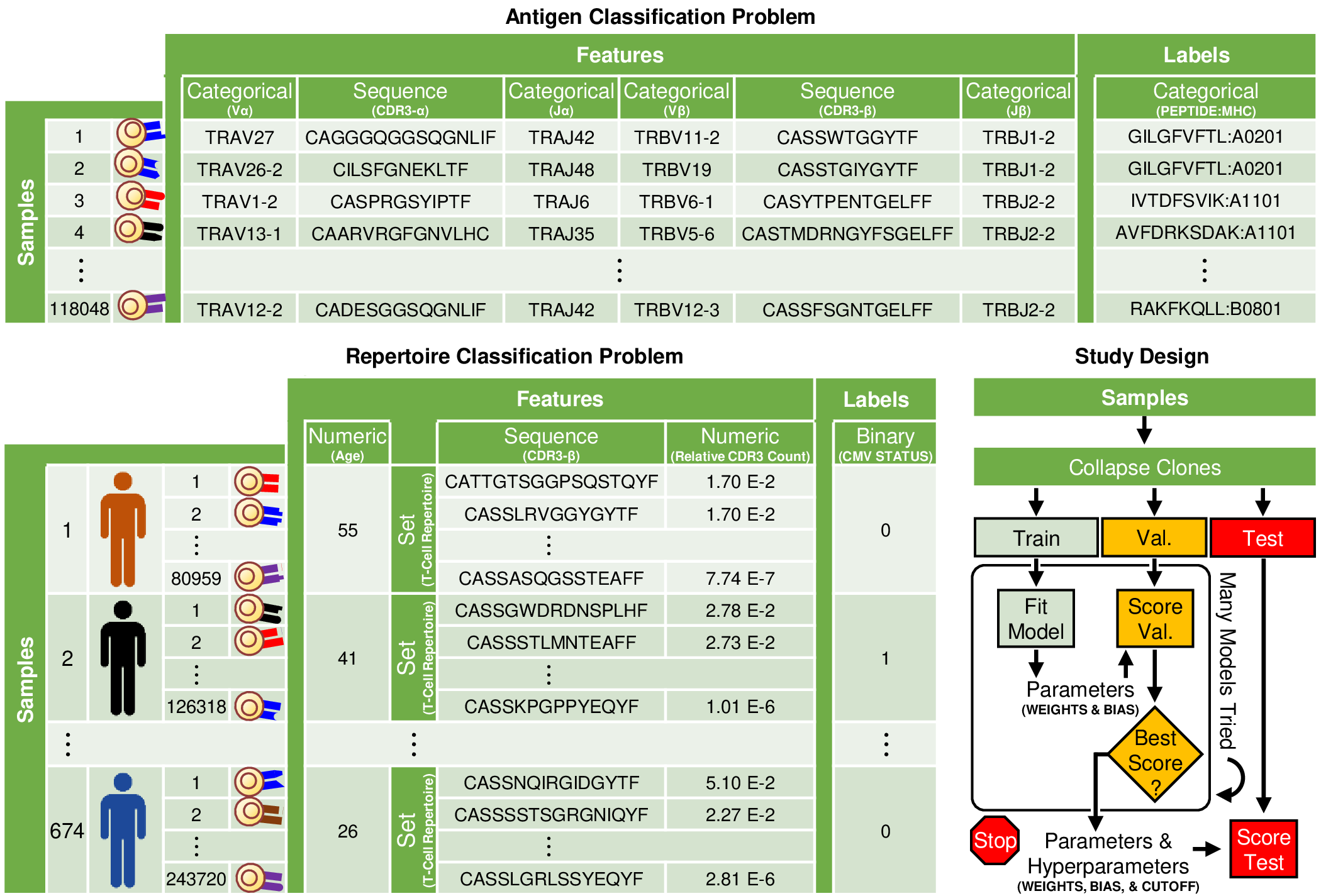

Supplement: S1 Data — (ZIP) [file pone.0265313.s009.zip › source code/artwork/data.png]

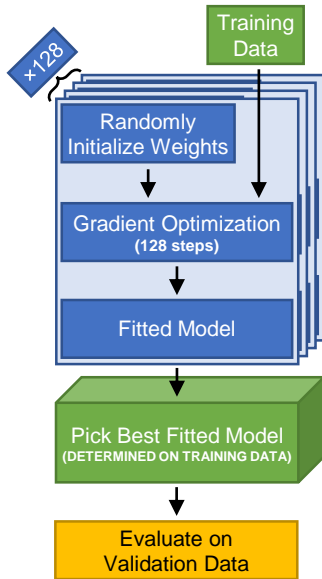

Supplement: S1 Data — (ZIP) [file pone.0265313.s009.zip › source code/artwork/many-fits.pdf]

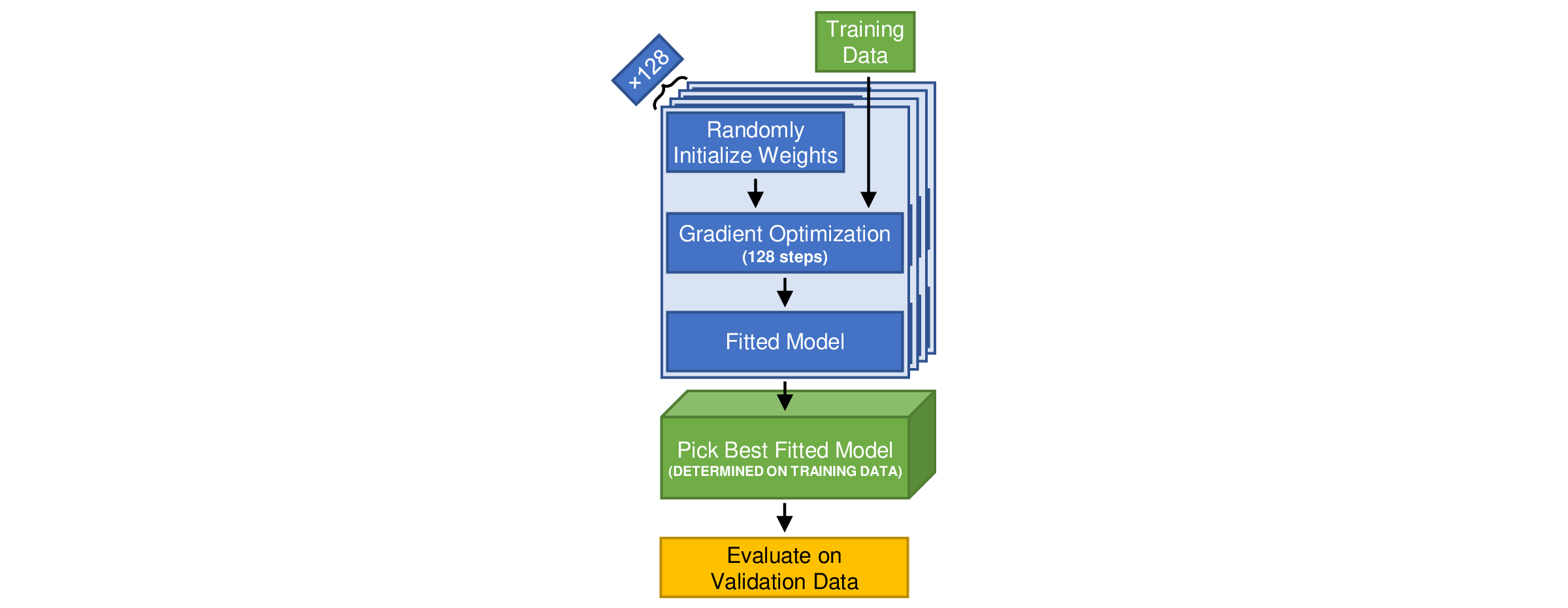

Supplement: S1 Data — (ZIP) [file pone.0265313.s009.zip › source code/artwork/many-fits.png]

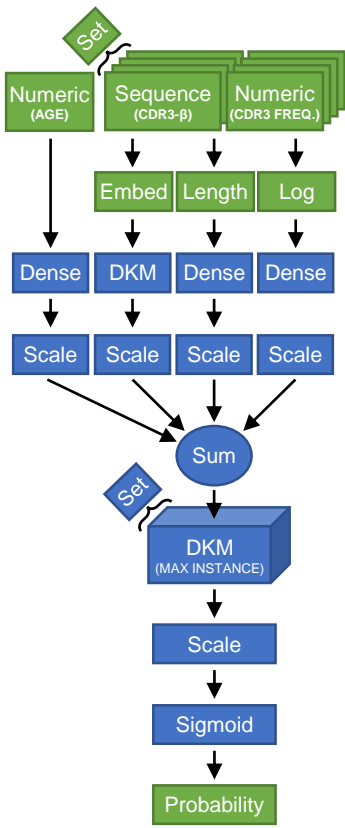

Supplement: S1 Data — (ZIP) [file pone.0265313.s009.zip › source code/artwork/repertoire-classification-model.pdf]

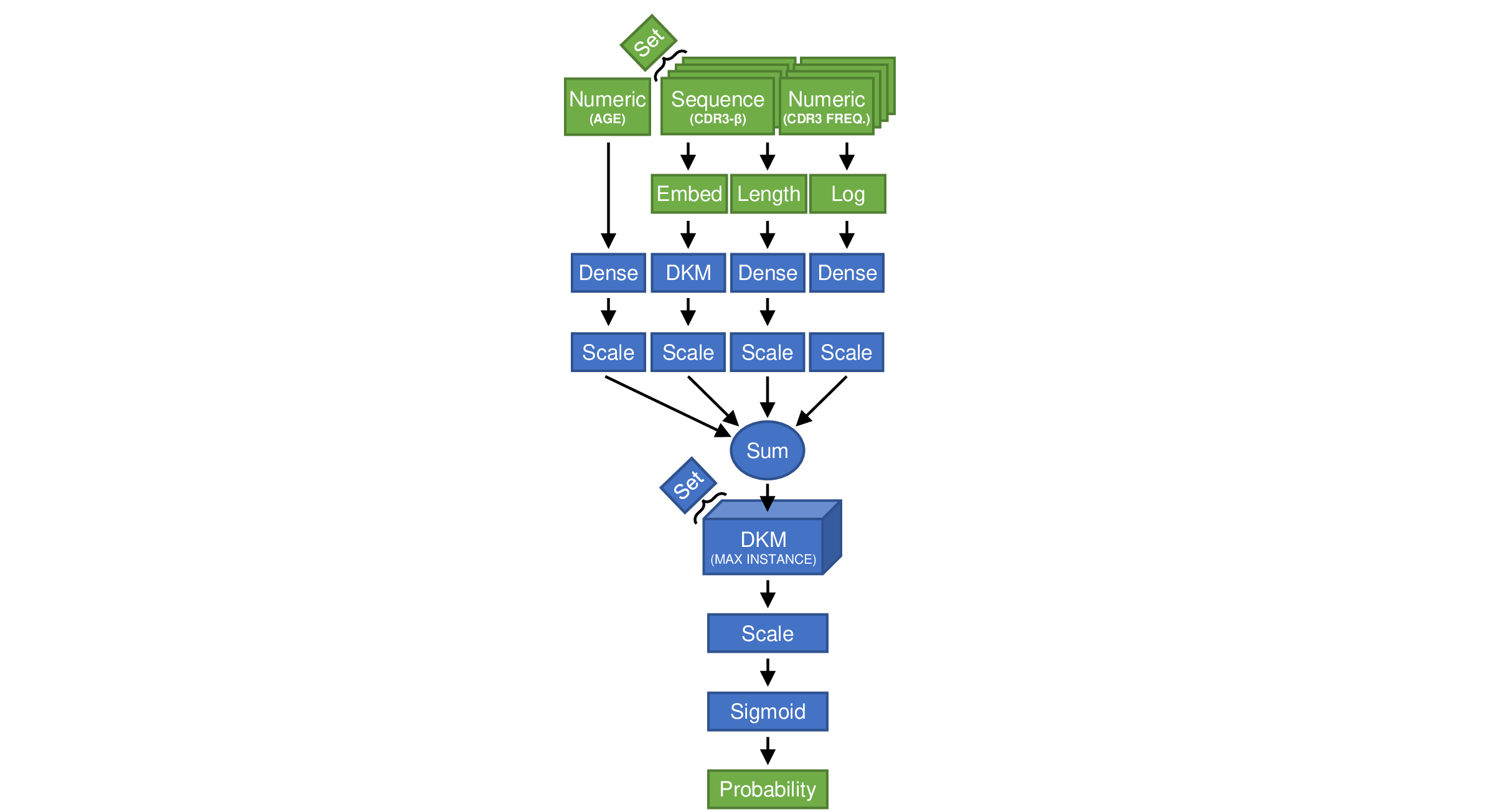

Supplement: S1 Data — (ZIP) [file pone.0265313.s009.zip › source code/artwork/repertoire-classification-model.png]
