# Supplementary material for: Dynamic kernel matching for non-conforming data: A case study of T cell receptor datasets
Source: PLoS One. 2023 Mar 7;18(3):e0265313. doi: 10.1371/journal.pone.0265313 (PMC9990938; doi:10.1371/journal.pone.0265313)
Supplement: S1 File — (DOCX) [file pone.0265313.s008.docx]

Dynamic Kernel Matching for Non-conforming Data: A Case Study of T-cell Receptor Datasets

**Supplementary Text**

Jared Ostmeyer, Lindsay Cowell, and Scott Christley

**Dynamic Kernel Matching *(Supplemental)***

*Discouraging Unmatched Symbols:* Algorithms for matching symbols may leave some symbols unmatched, which are represented as gaps by sequence alignment algorithms. To discourage unmatched symbols, we can introduce penalties incurred by the objective function of these algorithms whenever a symbol is left unmatched. For example, when $X$ is shorter than $\Theta$ and we want to ensure every symbol in $X$ is used, we can introduce a penalty $G_{x}$ approximating $-\infty$, constraining the alignment algorithm to use every symbol in $X$ to avoid the penalty. Alternatively, when $\Theta$ is shorter than $X$ and we want to ensure every symbol in $\Theta$ is used, we can introduce a penalty $G_{\theta}$ approximating $-\infty$, constraining the alignment algorithm to use every symbol in $\Theta$ to avoid the penalty.

*Data Splits:* On the antigen classification problem, identical TCR sequences are collapsed before splitting the TCR sequences into a training, validation, and test cohort using a 60/20/20 split. On the repertoire classification problem, the patients are separated into Cohort I and Cohort II. Patients in Cohort I are randomly shuffled. 120 patients in Cohort I are set aside for the validation cohort while the remaining patients in Cohort I are used as the training cohort. Patients in Cohort II are used as the test cohort.

*Data Balancing:* The dataset for the antigen classification problem is highly imbalanced because some pMHCs interact with significantly more TCRs than others. Sample frequencies $f_{j}$ assigned to each TCR balance the expected observation of each pMHC to $1/M_{\text{pMHC}}$, where $M_{\text{pMHC}}$ denotes the number of pMHCs.

$$\begin{aligned} \begin{matrix} f_{j}^{\left( i \right)}=\frac{c_{j}^{(i)}}{\sum_{j^{'}} c_{j'}^{(i)}} & f_{j}=\frac{1}{M_{\text{pMHC}}}\sum_{i=1}^{M_{\text{pMHC}}} f_{j}^{\left( i \right)} \end{matrix} \end{aligned}$$

Here, $c_{j}^{(i)}$ is the number of cases where the $j$^th^ TCR interacts with the $i$^th^ pMHC, $f_{j}^{\left( i \right)}$ is the frequency of the $j$^th^ TCR relative to all other TCRs interacting with the $i$^th^ pMHC, and $f_{j}$ is calculated as the average of $f_{j}^{\left( i \right)}$ across all pMHCs. Because a TCR can interact with multiple pMHCs, the observed probability of interacting with the $i$^th^ pMHC is computed as $y_{j}^{(i)}={c_{j}^{(i)}}/{\sum_{i^{'}=1}^{M_{\text{pMHC}}} c_{j}^{(i')}}$, which serves as the labels for this dataset.

The dataset for the repertoire classification problem is also imbalanced because a slightly greater number of patients are CMV+. Sample frequencies $f_{j}$ assigned to each patient $j$ balance the expected observation of CMV+ to CMV-.

$$f_{j}=\frac{1}{2}\cdot\left\{ \begin{aligned} \begin{matrix} \frac{1}{M_{\text{CMV}+}} & \text{for CMV+ patients} \end{matrix} \\ \begin{matrix} \frac{1}{M_{\text{CMV}-}} & \text{for CMV- patients} \end{matrix} \end{aligned} \right.$$

$M_{\text{CMV}+}$ and $M_{\text{CMV}-}$ are the number of patients with positive and negative CMV serostatus, respectively (different values for $M_{\text{CMV}+}$ and $M_{\text{CMV}-}$ are calculated for the training, validation, and test cohorts).

*Mixing Conforming and Non-conforming Features:* Both the antigen and repertoire classification datasets contain conforming and non-conforming features that can be used in conjunction to classify each sample (Fig. 1c,d). For both datasets, separate weights are assigned for conforming and non-conforming features. The dot product of the conforming features and weights is computed separately and added to the dot product of the non-conforming features and weights computed using equation (2). The sum of the dot products represents the dot product of weights and combined features.

*Scaling:* The dot product between the features and weights are computed piecewise for each group of features. For example, the dot product of the Vβ -gene features and associated weights is computed separately from the dot product of the CDR3-β features and associated weights. The weights of each feature group are scaled such that the dot product will have unit variance and zero mean, balancing the expected contribution from each feature group thereby implementing a naïve strategy of assigning equal importance to each feature group. The dot products from each feature group are added together to produce a dot product of all the features with all the weights. The weights are then scaled again to ensure the final dot product has unit variance and zero mean.

Scaling the weights is achieved using a procedure like batch normalization [4].

$$\begin{aligned} \begin{matrix} \mu=\sum_{j} f_{j}\cdot l_{j} & \sigma^{2}=\sum_{j} f_{j}\cdot\left( l_{j}-\mu_{j} \right)^{2} & l_{j}^{'}=\frac{l_{j}-\mu}{\sqrt{\sigma^{2}}} \end{matrix}\#\left( 1 \right) \end{aligned}$$

For any feature group, $l_{j}$ represents the value of the dot product (or output) from the $j$^th^ sample. The sample frequencies $f_{j}$ weight the calculations (as defined in the previous section). The mean $\mu$ and variance $\sigma^{2}$ are calculated over the training dataset immediately after initializing the weights but before gradient optimization. Once $\mu$ and $\sigma^{2}$ are calculated they can be refactored into the weights and bias values (or left as an additional calculation after each dot product). This scaling operation is conceptually like batch normalization but applied over the entire dataset (not just a batch), weighted by sample frequency, and where $\mu$ and $\sigma^{2}$ are not updated during optimization.

*Similarities Between the Alignment Score and Max-Pooling:* Both DKM and convolutional neural networks (CNNs) generate predictions from maximal responses. The alignment score in DKM represents the maximum possible sum of similarity scores between $x_{i}$ and $\theta_{j}$, whereas the pooling layer of CNNs represents the maximum possible responses from the convolutional layer. The reason why the alignment score works for classifying non-conforming features may be like the reason why pooling works in CNNs. The similarity of DKM with CNNs suggests that we can swap an alignment algorithm for each max-pooling operation in a multi-layer CNN to create a deep DKM neural network for classifying non-conforming features.

*Loss Function:* Numbers for the weights and bias are picked to minimize a loss function. We use the KL-divergence as the loss function, which measures the number of extra computer bits required to encode the labels when the predictions are used instead of the labels.

$$D_{KL}=-\sum_{j} f_{j}\cdot\sum_{i=1}^{M} y_{j}^{(i)}\cdot\ln\frac{p_{j}^{(i)}}{y_{j}^{(i)}}$$

$D_{KL}$ represents the KL divergence. The smaller the number the better the fit, with $0$ representing the best fit possible. The outer summation is over all samples in the datasets, indexed by $j$. Each sample is weighted by its frequency $f_{j}$, representing the relative number of times that sample appears in the dataset. The inner summation is over the label categories. $M$ represents the number of label categories and $i$ indicates a specific label category. For the antigen classification problem, $M=6$ and for the repertoire classification problem $M=2$. The labels (ground truth) for each category are represented by $y_{j}^{(i)}$ and the probabilities assigned to each category by the statistical classifier are represented by $p_{j}^{(i)}$.

*Optimization:* We use adam, a gradient optimization-based technique, to minimize the loss function, referred to as the *fit*, over a cohort of samples used for training the statistical classifier [5]. To start, the weights are randomly *initialized*, which is where random numbers are assigned as the initial values for the weights. We use distributions described by Glorot and Bengio when drawing these random numbers [6]. Next, the optimization routine is repeatedly run for many steps. At every step, (i) equation (1) is used to compute the similarity scores, (ii) symbols are matched to maximize the alignment score as described, (iii) the alignment score is treated as a sum of features multiplied by weights, like in equation (2), (iv) a prediction is made, (v) the loss function is calculated by comparing predictions to labels, (vi) the negative of the gradient of the loss function with respect to the weights is computed, and (vii) the direction and magnitude of the gradients are used to make small changes to the weights to reduce the loss function. At each step, we must rerun i through vii.

Gradient optimization techniques do not guarantee finding the globally optimal solution, which is why we refit each statistical classifier multiple times, attempting to verify the best fit. With each refit, the weights are re-initialized before repeatedly running the optimization routine, allowing us to check that the fit does not depend on the initial conditions. When the fits vary considerably, we take the best fit *as measured over samples in the training cohort*, representing our attempt to find the global optimal fit (Supplementary Fig. 5j). Only the weights from the best fit are then used when evaluating samples from the validation and test cohorts.

*Avoiding Overfitting:* With DKM, weights are assigned to features based on a similarity score rather than having a dedicated weight for each feature, decoupling the number of weights from the number of features. In this study, we pick the number of weights to be an order of magnitude less than the number of samples in the training cohort, reducing the risk of overfitting, a phenomenon where a statistical classifier fails to generalize to holdout samples.

**Results *(Supplemental)***

*Permutation Analysis on the Antigen Classification Problem:* As a control, we permute the features with respect to the labels, eliminating any relationship between the features and labels, and refit the statistical classifier to verify that it no longer generalizes to holdout samples. On permuted data, the fit to samples from the training cohort steadily improves from 2.65 to 1.18 bits, representing the statistical classifier’s capacity to memorize labels when there is no relationship between the features and labels. The fit as measured to samples from the validation cohort worsens from 2.91 to 3.14 bits, worse than the 2.54 bits that would be considered significant, indicating no ability to generalize to holdout samples when the statistical classifier memorizes labels. The fit to the test cohort under permutation is 3.17 bits, consistent with results from the validation cohort under permutation.

*Permutation Analysis on the Repertoire Classification Problem:* As a control, we permute the features with respect to the labels, eliminating any relationship between the features and labels, and refit the statistical classifier to verify that it no longer generalizes to holdout samples. On permuted data, the fit to samples from the training cohort steadily improves from 1.09 to 0.927 bits, representing the statistical classifier’s capacity to memorize labels when there is no relationship between the features and labels. The fit as measured over samples from the validation cohort fluctuates between 1.18 to 1.016 bits, worse than the 1.0 bit that would be considered significant, indicating no ability to generalize to holdout samples when the statistical classifier memorizes labels. The fit on samples from the test cohort under permutation is 1.07 bits, consistent with results from the validation cohort under permutation.

*Other Variations of DKM on the Repertoire Classification Problem:* To create a statistical classifier to handle the set of sequences, we represent the symbols in $\Theta$ as a set of sequences (see *Matching Features from a Set of Sequences to Weights* for the definition of sequences in $\Theta$). For $\Theta$, we need to pick the number of sequences in the set using our understanding of the problem to make an appropriate selection. Therefore, we turn to our understanding of immunology. In typical immune responses, only a handful of T-cells express a receptor that can bind the underlying pMHC. The remaining T-cells, representing most of the repertoire, are bystanders. To constrain the statistical classifier to identify the single most relevant TCR, ignoring receptors from bystander T-cells, we pick to have only one sequence in the set for $\Theta$. We solve the assignment problem by brute force to identify the single most relevant CDR3 sequence in the TCR repertoire to match with the single sequence in the set for $\Theta$.

In Supplementary Fig. 6, we show the result with this constraint relaxed, picking to have two sequences in the set for $\Theta$. We again solve the assignment problem by brute force to identify two relevant CDR3 sequences in the TCR repertoire to match the two sequences in the set of $\Theta$. With this added capacity, we observe the performance of the statistical classifier marginally improving.

*Analyzing Patient Age as a Feature on the Repertoire Classification Problem:* Because elderly populations are more vulnerable to CMV infection and it is well known that there is a correlation between age and CMV positivity, we wondered if the statistical classifier is using each patient’s age to predict their CMV serostatus. We included patient age to capture this effect in our model. Because of the simplicity of our model, we can look at the weight on the patient age to determine the importance of this feature in the model’s predictions. Surprisingly, the weight for the patient age has a value of 0.00938 or almost zero. By comparison, the average magnitude for the weights on the Atchley factors used to represent each amino acid residue is 0.212. Therefore, patient age makes no practical contribution in the predictions of our statistical classifier. We do not mean to imply that age does not predict CMV serostatus, only that our statistical classifier was unable to uncover age as a predictor of CMV serostatus from the dataset used in this study.

*Capturing Additional Samples with Confidence Cutoffs:* To capture additional samples, we can remove samples captured by the confidence cutoff, which are classified with an accuracy ≥95%, and refit the statistical classifier to the uncaptured samples, using the refitted model to capture additional samples with a classification accuracy of ≥95%. We can continue repeating this process to capture additional samples with an accuracy of ≥95% until no more samples can be captured with this degree of accuracy. The approach resembles boosting, where multiple weak statistical classifiers are combined into a single, stronger statistical classifier. We anticipate our future studies will combine DKM augmented statistical classifiers with boosting methods, like described, achieving higher classification accuracies over a larger subset of the samples.

# **References**

| [1] | J. Ostmeyer, S. Christley, W. H. Rounds, I. Toby, B. M. Greenberg, N. L. Monson and L. G. Cowell, "Statistical classifiers for diagnosing disease from immune repertoires: a case study using multiple sclerosis.," *BMC Bioinformatics,* vol. 18, no. 1, p. 401, 2017. |
| --- | --- |
| [2] | J. Ostmeyer, S. Christley, I. T. Toby and L. G. Cowell, "Biophysicochemical motifs in T-cell receptor sequences distinguish repertoires from tumor-infiltrating lymphocyte and adjacent healthy tissue," *Cancer Research,* vol. 79, no. 7, pp. 1671-1680, 2019. |
| [3] | J. L. Ostmeyer, L. G. Cowell and S. Christley, "Developing and validating an approach for diagnosing and prognosticating cancer from biochemical motifs in T-cell receptors.," *Journal of Clinical Oncology,* vol. 38, 2020. |
| [4] | S. Ioffe and C. Szegedy, "Batch Normalization: Accelerating Deep Network Training by Reducing Internal Covariate Shift," in *Proceedings of The 32nd International Conference on Machine Learning*, 2015. |
| [5] | D. P. Kingma and J. L. Ba, "Adam: A Method for Stochastic Optimization," in *ICLR 2015 : International Conference on Learning Representations 2015*, 2015. |
| [6] | X. Glorot and Y. Bengio, "Understanding the difficulty of training deep feedforward neural networks," in *Proceedings of the Thirteenth International Conference on Artificial Intelligence and Statistics*, 2010. |
