# Supplementary material for: Dynamic kernel matching for non-conforming data: A case study of T cell receptor datasets
Source: PLoS One. 2023 Mar 7;18(3):e0265313. doi: 10.1371/journal.pone.0265313 (PMC9990938; doi:10.1371/journal.pone.0265313)
Supplement: S1 Data — (ZIP) [file pone.0265313.s009.zip › source code/artwork/antigen-classification-model.pptx]

## Slide 1
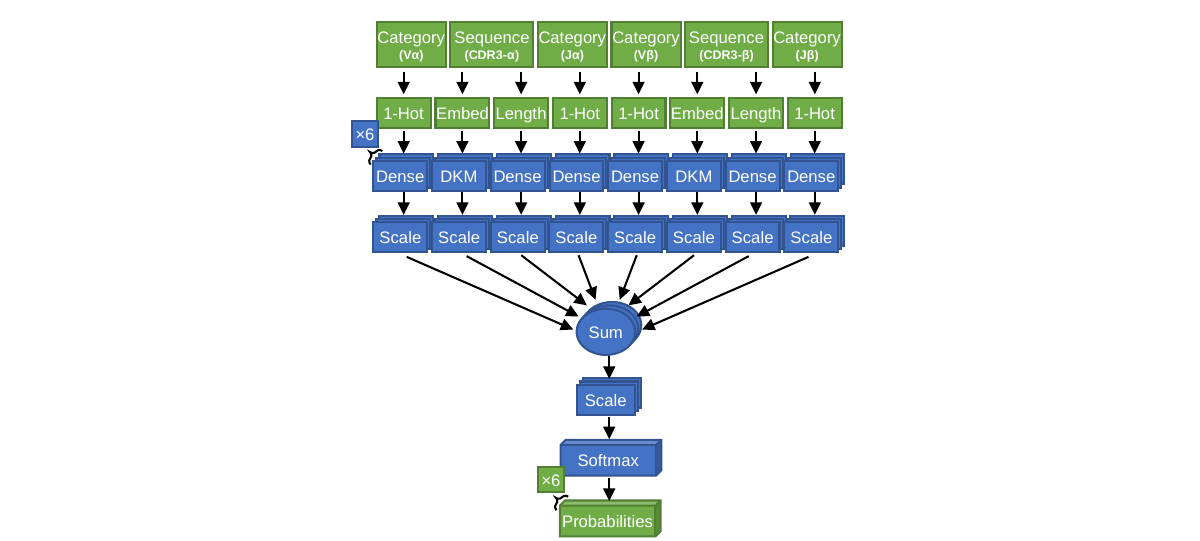

Category
(Vα)
Sequence
(CDR3-α)
Category
(Jα)
Category
(Vβ)
Sequence
(CDR3-β)
Category
(Jβ)
1-Hot
Embed
Length
1-Hot
1-Hot
Embed
Length
1-Hot
×6
Dense
DKA
Dense
Dense
Dense
DKA
Dense
Dense
Scale
Scale
Scale
Scale
Scale
Scale
Scale
Scale
Sum
Norm
Dense
DKA
Dense
Dense
Dense
DKA
Dense
Dense
Scale
Scale
Scale
Scale
Scale
Scale
Scale
Scale
Sum
Norm
Dense
DKM
Dense
Dense
Dense
DKM
Dense
Dense
Scale
Scale
Scale
Scale
Scale
Scale
Scale
Scale
Sum
Scale
Softmax
×6
Probabilities
