# Supplementary material for: Dynamic kernel matching for non-conforming data: A case study of T cell receptor datasets
Source: PLoS One. 2023 Mar 7;18(3):e0265313. doi: 10.1371/journal.pone.0265313 (PMC9990938; doi:10.1371/journal.pone.0265313)

Antigen Classification Problem

Samples	Categorical (Vα)	Sequence (CDR3-α)	Categorical (Jα)	Categorical (Vβ)	Sequence (CDR3-β)	Categorical (Jβ)	Labels: Categorical (PEPTIDE:MHC)
	**Features**						**Labels**
1	TRAV27	CAGGGQGGSQGNLIF	TRAJ42	TRBV11-2	CASSWTGGYTF	TRBJ1-2	GILGFVFTL:A0201
2	TRAV26-2	CILSFGNEKLTF	TRAJ48	TRBV19	CASSTGIYGYTF	TRBJ1-2	GILGFVFTL:A0201
3	TRAV1-2	CASPRGSYIPTF	TRAJ6	TRBV6-1	CASYTPENTGELFF	TRBJ2-2	IVTDFSVIK:A1101
4	TRAV13-1	CAARVRGFGNVLHC	TRAJ35	TRBV5-6	CASTMDRNGYFSGELFF	TRBJ2-2	AVFDRKSDAK:A1101
⋮			⋮				⋮
118048	TRAV12-2	CADESGGSQGNLIF	TRAJ42	TRBV12-3	CASSFSGNTGELFF	TRBJ2-2	RAKFKQLL:B0801

Repertoire Classification Problem

Samples		Numeric (Age)	Set (T-Cell Repertoire)	Sequence (CDR3-β)	Numeric (Relative CDR3 Count)	Labels: Binary (CMV STATUS)
		**Features**				**Labels**
1	1	55	Set (T-Cell Repertoire)	CATTGTSGGPSQSTQYF	1.70 E-2	0
	2			CASSLRVGGYGYTF	1.70 E-2	
	⋮			⋮		
	80959			CASSASQGSSTEAFF	7.74 E-7	
2	1	41	Set (T-Cell Repertoire)	CASSGWDRDNSPLHF	2.78 E-2	1
	2			CASSSTLMNTEAFF	2.73 E-2	
	⋮			⋮		
	126318			CASSKPGPPYEQYF	1.01 E-6	
⋮		⋮				⋮
674	1	26	Set (T-Cell Repertoire)	CASSNQIRGIDGYTF	5.10 E-2	0
	2			CASSSSTSGRGNIQYF	2.27 E-2	
	⋮			⋮		
	243720			CASSLGRLSSYEQYF	2.81 E-6	

Study Design

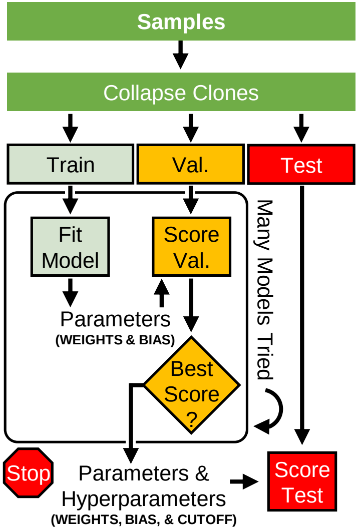

Supplement: S1 Data — (ZIP) [file pone.0265313.s009.zip › source code/artwork/data.pdf]
