# Supplementary material for: Dynamic kernel matching for non-conforming data: A case study of T cell receptor datasets
Source: PLoS One. 2023 Mar 7;18(3):e0265313. doi: 10.1371/journal.pone.0265313 (PMC9990938; doi:10.1371/journal.pone.0265313)
Supplement: S1 Data — (ZIP) [file pone.0265313.s009.zip › source code/artwork/data.pptx]

## Slide 1
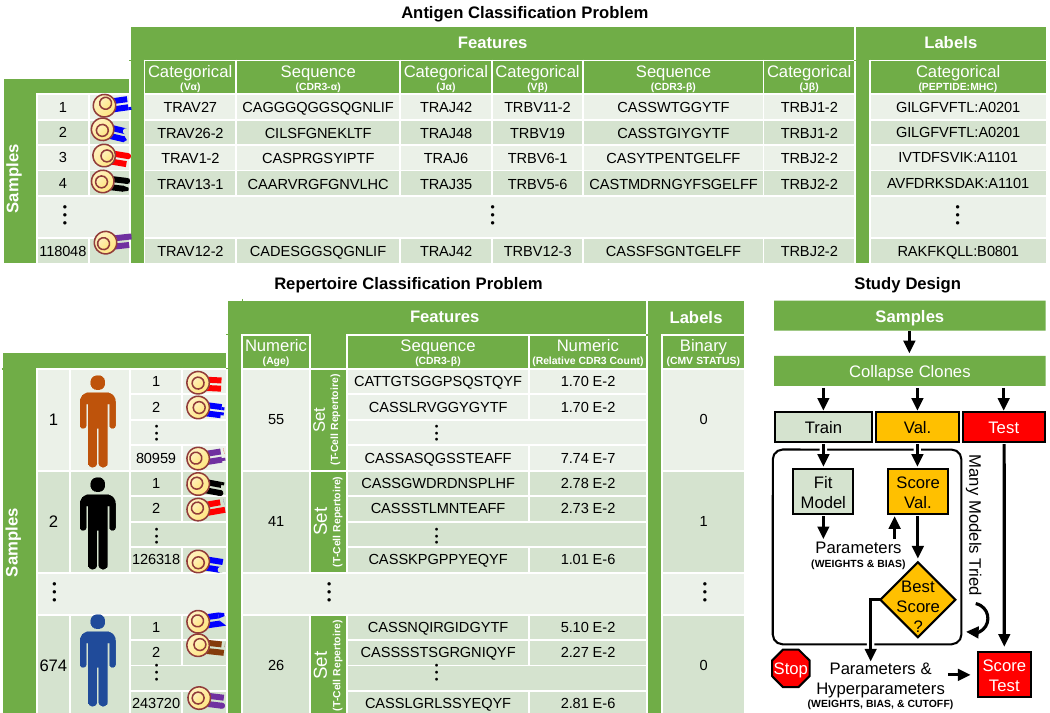

Antigen Classification Problem
| | | | Features | | | | | | | Labels | |
| --- | --- | --- | --- | --- | --- | --- | --- | --- | --- | --- | --- |
| | | | -- | Categorical (Vα) | Sequence (CDR3-α) | Categorical (Jα) | Categorical (Vβ) | Sequence (CDR3-β) | Categorical (Jβ) | -- | Categorical (PEPTIDE:MHC) |
| | | | | | | | | | | | |
| Samples | 1 | -------- | | TRAV27 | CAGGGQGGSQGNLIF | TRAJ42 | TRBV11-2 | CASSWTGGYTF | TRBJ1-2 | | GILGFVFTL:A0201 |
| | 2 | | | TRAV26-2 | CILSFGNEKLTF | TRAJ48 | TRBV19 | CASSTGIYGYTF | TRBJ1-2 | | GILGFVFTL:A0201 |
| | 3 | | | TRAV1-2 | CASPRGSYIPTF | TRAJ6 | TRBV6-1 | CASYTPENTGELFF | TRBJ2-2 | | IVTDFSVIK:A1101 |
| | 4 | | | TRAV13-1 | CAARVRGFGNVLHC | TRAJ35 | TRBV5-6 | CASTMDRNGYFSGELFF | TRBJ2-2 | | AVFDRKSDAK:A1101 |
| | | | | | | | | | | | |
| | 118048 | | | TRAV12-2 | CADESGGSQGNLIF | TRAJ42 | TRBV12-3 | CASSFSGNTGELFF | TRBJ2-2 | | RAKFKQLL:B0801 |
• • •
• • •
• • •
Repertoire Classification Problem
Study Design
| | | | | | | Features | | | | Labels | |
| --- | --- | --- | --- | --- | --- | --- | --- | --- | --- | --- | --- |
| | | | | | | Numeric (Age) | | Sequence (CDR3-β) | Numeric (Relative CDR3 Count) | -- | Binary (CMV STATUS) |
| | | | | | | | | | | | |
| Samples | 1 | ---------- | 1 | -------- | | 55 | Set (T-Cell Repertoire) | CATTGTSGGPSQSTQYF | 1.70 E-2 | | 0 |
| | | | 2 | | | | | CASSLRVGGYGYTF | 1.70 E-2 | | |
| | | | | | | | | | | | |
| | | | 80959 | | | | | CASSASQGSSTEAFF | 7.74 E-7 | | |
| | 2 | | 1 | | | 41 | Set (T-Cell Repertoire) | CASSGWDRDNSPLHF | 2.78 E-2 | | 1 |
| | | | 2 | | | | | CASSSTLMNTEAFF | 2.73 E-2 | | |
| | | | | | | | | | | | |
| | | | 126318 | | | | | CASSKPGPPYEQYF | 1.01 E-6 | | |
| | | | | | | | | | | | |
| | 674 | | 1 | | | 26 | Set (T-Cell Repertoire) | CASSNQIRGIDGYTF | 5.10 E-2 | | 0 |
| | | | 2 | | | | | CASSSSTSGRGNIQYF | 2.27 E-2 | | |
| | | | | | | | | | | | |
| | | | 243720 | | | | | CASSLGRLSSYEQYF | 2.81 E-6 | | |
Samples
Collapse Clones
Val.
Train
Test
• • •
• • •
Fit Model
Score Val.
Many Models Tried
• • •
• • •
Parameters
(WEIGHTS & BIAS)
Best
Score
?
• • •
• • •
• • •
Stop
Parameters & Hyperparameters
(WEIGHTS, BIAS, & CUTOFF)
Score
Test
• • •
• • •
