# Supplementary material for: Dynamic kernel matching for non-conforming data: A case study of T cell receptor datasets
Source: PLoS One. 2023 Mar 7;18(3):e0265313. doi: 10.1371/journal.pone.0265313 (PMC9990938; doi:10.1371/journal.pone.0265313)
Supplement: S1 Data — (ZIP) [file pone.0265313.s009.zip › source code/artwork/many-fits.pptx]

## Slide 1
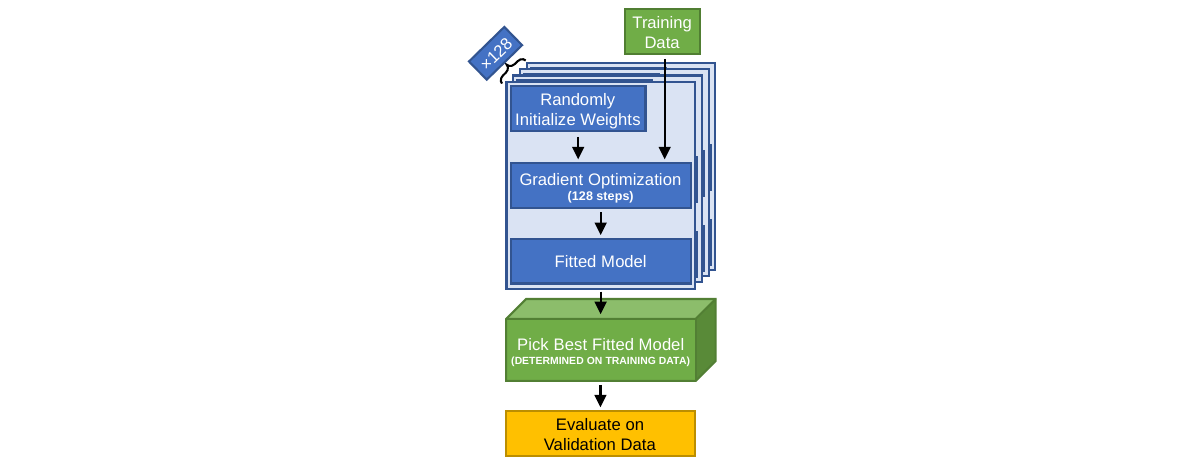

Training Data
×128
Randomly Initialize Weights
Gradient Optimization
(1024 steps)
Fitted Model
Randomly Initialize Weights
Gradient Optimization
(1024 steps)
Fitted Model
Randomly Initialize Weights
Gradient Optimization
(1024 steps)
Fitted Model
Randomly Initialize Weights
Gradient Optimization
(128 steps)
Fitted Model
Pick Best Fitted Model
(DETERMINED ON TRAINING DATA)
Evaluate on Validation Data
