# Supplementary material for: Dynamic kernel matching for non-conforming data: A case study of T cell receptor datasets
Source: PLoS One. 2023 Mar 7;18(3):e0265313. doi: 10.1371/journal.pone.0265313 (PMC9990938; doi:10.1371/journal.pone.0265313)
Supplement: S1 Data — (ZIP) [file pone.0265313.s009.zip › source code/artwork/repertoire-classification-model.pptx]

## Slide 1
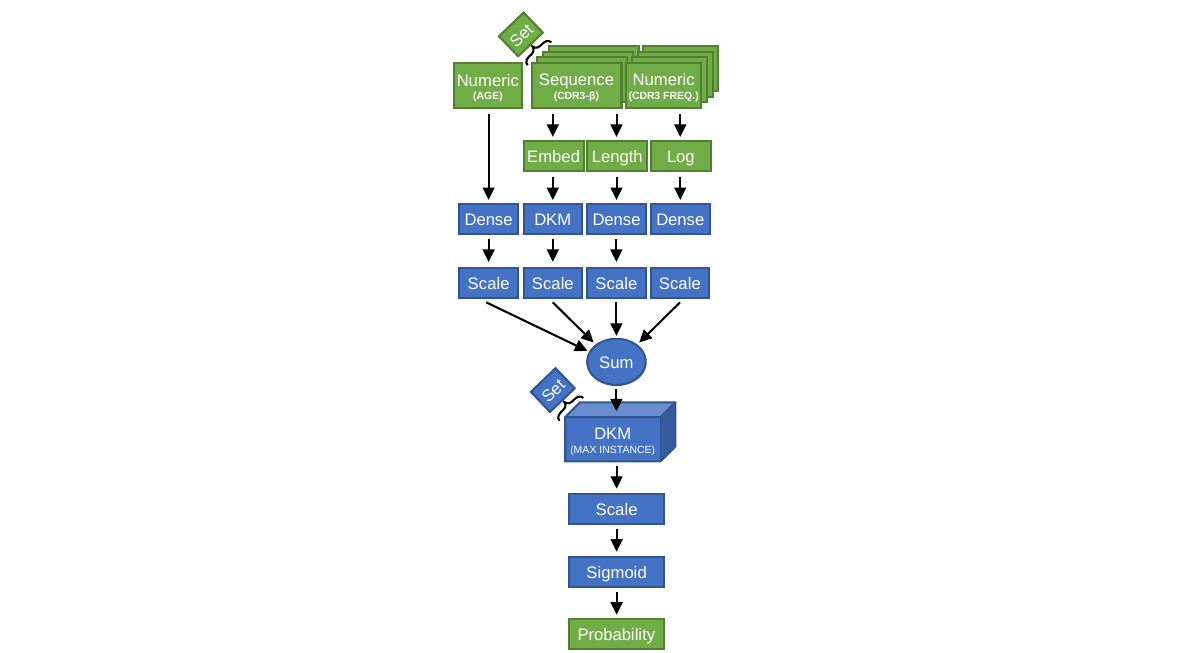

Set
Sequence
(CDR3-α)
Numeric
(Cell Freq.)
Sequence
(CDR3-α)
Numeric
(Cell Freq.)
Sequence
(CDR3-α)
Numeric
(Cell Freq.)
Sequence
(CDR3-β)
Numeric
(CDR3 FREQ.)
Numeric
(AGE)
Dense
Scale
Embed
Length
Log
DKM
Dense
Dense
Scale
Scale
Scale
Sum
Set
DKM
(MAX INSTANCE)
Scale
Sigmoid
Probability
