# Supplementary material for: Dynamic kernel matching for non-conforming data: A case study of T cell receptor datasets
Source: PLoS One. 2023 Mar 7;18(3):e0265313. doi: 10.1371/journal.pone.0265313 (PMC9990938; doi:10.1371/journal.pone.0265313)
Supplement: Attachment — Submitted filename: response-3_20211022e.docx [file pone.0265313.s010.docx]

Dynamic Kernel Matching for Non-conforming Data: A Case Study of T Cell Receptor Datasets

**Response to Reviewers**

**Reviewer #3:** In the line 258, the author told us “there were 44 pMHCs in the database, while only the six pMHCs could interacted with the receptors”, how about the other 38 pMHCs? Maybe some of the them could be used for the antigen classification, but the accuracy of the algorithm in not high enough to predict them, or the author should use the other methods, such as the biological experimental, to verified the other 38 pMHCs had not been used for antigen classification.

**Response:** For the antigen classification problem, all the models have exactly six outputs, one for each pMHC. For this reason, our comparisons of DKM to other models do not generalize beyond these six pMHCs. Given this constraint, we cannot utilize the remaining 38 pMHCs to further compare DKM to other approaches.

To train a model to *categorize* each TCR by pMHC, examples of TCRs interacting with each pMHC are required. Of the 44 pMHCs in the dataset, only the six pMHCs interacting with at least 500 unique receptors are used *because* only these six pMHCs provide enough examples to train a categorical model. With a minimum of 500 TCRs per pMHC, we could split the TCRs into a training, validation, and test cohort.

I initially considered a generative model *(as opposed to a categorical model)* that could predict the interactions of any TCR with any pMHC. However, my initial analyses determined any generative model would require far more than the 44 pMHC examples available in the antigen classification dataset. As more pMHCs become available in the future, we will reevaluate if we can construct a generative model for all pMHCs.

**Reviewer #3:** In line 371, “the classification accuracy of DKM is significantly worse than Emerson’s method on the repertoire classification problem”, in author’s opinion, they thought the advantage of DKM was that it could predict the antigen classification and repertoire classification simultaneously, but the prediction results of repertoire would definitely affect the subsequent results. Furthermore, if the time complexity was not high enough, other researchers could run the two different models to obgtain the classification results of antigen and repertoire, to replace the DKM. Thus, to some extent, the author should modified the DKM model, such as some parameters or others, to improve the accuracy in predicting antigen and repertoire classification.

**Response:** In response to your suggestion, I added another hyperparameter for the DKM model on the repertoire classification task. For this hyperparameter, I experimented with trimming CDR3 sequences because previous studies have shown the beginning and end of the CDR3 sequence are not involved in antigen recognition [1,2]. Unfortunately, trimming CDR3 sequences did not improve performance (supplementary figure 10).

For a list of various DKM models considered in this study, refer to supplementary figure 10. Some of the DKM variations performed better than the original model as described in the manuscript, but none of the models outperformed the original results reported by Emerson et al. I left the discussion of DKM variants in supplementary material because I had already unblinded us to the test cohort, so I did not feel comfortable reporting these results in the main manuscript.

I am unable to perform an exhaustive hyperparameter search because my resources are allocated by funded projects.

**Reviewer #3:** When compared the DKM model with other methods, in addition to the prediction accuracy, the author should use more indicators to explained them comprehensively.

**Response:** In response to your suggestion, we added area under the curve (AUC) for plots of the true versus false positive rates of each model. The AUC indicates how much the true positive rate is better than the false positive rate. An AUC of 1 indicates perfect predictive performance whereas an AUC of 0.5 indicates no predictive performance. The AUC is an excellent metric for measuring predictive performance on imbalanced datasets like ours, which is why I selected this metric. Because the AUC compares the true to false positive rates, it is designed for binary classifiers. To apply the AUC to a multinomial model with 6 outputs, we treated each output as a binary classifier using a one-vs-rest criteria, like others have done, by averaging the AUC for all 6 outputs [3].

On the antigen classification problem, our DKM model achieves an AUC of 0.91 (see supplementary figure 6), outperforming all other approaches. Our DKM model achieves the highest AUC of all the models for the antigen classification task. Indeed, our DKM model achieves the best performance across the three standard metrics we considered *(KL-divergence, balanced classification accuracy, and now AUC)*.

On the repertoire classification problem, our DKM model achieves an AUC of 0.75 (see supplemental figures 9, 10). While an AUC of 0.75 is okay, it is still below the AUC of 0.94 reported by Emerson et al.

In addition to the AUC, we report the KL-divergence *(or just the log-likelihood when appropriate)* and balanced classification accuracy, providing three standard metrics for interpreting performance. If there is a specific metric you would like us to consider, please suggest it to us in your response. Bear in mind that many metrics are limited to a single type of statistical classifier and do not generally apply to both binary and multinomial predictors.

Thank you!

Jared

**References:**

1. Ostmeyer, Jared, et al. "Biophysicochemical motifs in T-cell receptor sequences distinguish repertoires from tumor-infiltrating lymphocyte and adjacent healthy tissue." Cancer research 79.7 (2019): 1671-1680.
2. Glanville, Jacob, et al. "Identifying specificity groups in the T cell receptor repertoire." Nature 547.7661 (2017): 94-98.
3. De Neuter, Nicolas, et al. "On the feasibility of mining CD8+ T cell receptor patterns underlying immunogenic peptide recognition." Immunogenetics 70.3 (2018): 159-168.
