# Supplementary material for: Dynamic kernel matching for non-conforming data: A case study of T cell receptor datasets
Source: PLoS One. 2023 Mar 7;18(3):e0265313. doi: 10.1371/journal.pone.0265313 (PMC9990938; doi:10.1371/journal.pone.0265313)
Supplement: Attachment — Submitted filename: responses_20220114.docx [file pone.0265313.s011.docx]

**Reviewer #4:** This is an innovative study, described an approach (named dynamic kernel matching (DKM)) for modifying established statistical classifiers to handle non-conforming data. TCR sequences are one example of a datatype with non-conforming features. Two datasets of TCR sequences labelled by disease antigen and patient cytomegalovirus (CMV) serostatus were applied in the study. The statistical classifier performed well in the antigen classification dataset (the accuracy is 67.4%-70.5%). As for repertoire classification dataset, the accuracy is 67.6%, lower than a previously reported result by Emerson and colleagues. The author responded well to the reviewer#3’s queries.

Following are considerations for further improvement of the manuscript at the discretion of the authors and editors:

**Reviewer #4:** In the Methods & Materials, the author described the weights in DKM model. were the frequency of each TCR clone included in the weight calculation?

**Response:** The clonal frequencies are used indirectly to calculate the weights for the antigen and repertoire classification models. The clonal frequency is introduced into the calculations of the weights during the fitting procedure whenever we calculate a mean or standard deviation. We believe it is most appropriate to weight the terms in the mean and standard deviations by the clonal frequency. Means and standard deviations are calculated, for example, whenever we scale terms in the model using batch normalization *(see Supplementary Text, Dynamic Kernel Matching, Scaling and Supplemental Figures 3 and 7)*.

For the antigen classification problem, where each clone represents an individual sample, we use the clonal frequency to weight each sample in the loss function (objective function). This is an additional source by which the clonal frequency indirectly influences the calculation of the weights of the DKM model specific to the antigen classification model.

**Reviewer #4:**  How does the author homogenize and normalize the TCR sequences dataset of patient cytomegalovirus (CMV) serostatus? Especially, the TCR clones with low frequency could be sequencing errors.

**Response:** The numeric representations of the TCR sequences are passed to the model without being normalized. However, after multiplying each feature with a weight, we normalize the subsequent terms by batch normalization *(see Supplementary Text, Dynamic Kernel Matching, Scaling and Supplemental Figures 3 and 7)*. This ensures the expected magnitude of each term is the same at the start of the gradient optimization procedure. *Of note is that we wrote our own batch normalization code rather than use TensorFlow code because the existing code did not support weighted calculations of the mean and variance. To simplify our code for batch normalization, we only apply batch normalization at the start of but not during gradient optimization.*

While we expect some TCR sequences to contain a sequencing error, the vast majority will not. We ignore sequencing error when predicting the CMV serostatus because sequencing error effects only a small number of the features and not the label, which is based on serostatus.

**Reviewer #4:**  In the line 403, the author told us “For missing features, like patient age, we use a value of zero”, Does this affect the accuracy of the classifier? All patients’ age was number greater than 0, and age is an important factor affecting immunity.

**Response:** I failed to communicate what we are doing at line 403, so I rewrote that text to clarify what we did.

We were not attempting to fill in missing features for the repertoire classification problem *(the patient age is known for every sample)*. Rather, we were attempting to determine if a DKM model fitted to the repertoire classification problem is forced to learn how to identify TCR sequences with CMV specificity. To test this, we fit the DKM model to the repertoire classification problem and applied the weights to score individual TCR sequences with known specificity. However, some of the features in the repertoire classification problem, like patient age, are not features of the TCR sequences with known specificity. Therefore, we assigned all these features a value of 0, which is fine because we are trying to score individual TCRs rather than predict a label for an entire repertoire.

**Reviewer #4:**  The ROC curves of the test cohort could be put into the result of article.

**Response:** For the repertoire classification problem, I updated figure 3 to contain a ROC curve. For the antigen classification problem, I updated figure 2 with a confusion matrix because we treated the antigen classification problem as a multinomial classification problem. Alternatively, we could have treated the multinomial classifier as many separate binary classifiers and generate ROC curves for each binary classifier. Ultimately, we believed the reader would have a harder time interpreting a series of ROC curves generated from a single multinomial model, which is why we chose to go with a confusion matrix instead.

Thank you, Dr. Wang.
